# A 62-year-old Man with Acute Dizziness, Nausea and Vomiting

**DOI:** 10.22114/ajem.v0i0.193

**Published:** 2019-07-19

**Authors:** Amir Noyani, Hosein Sheidaey, Zeinab Mohammadi, Mahtab Hajian, Farangis Sadeghi

**Affiliations:** 1. Clinical Research Development Unit, Imam Hossein Hospital, Shahroud University of Medical Sciences, Shahroud, Iran.; 2. Student Research Committee, School of Medicine, Shahroud University of Medical Sciences, Shahroud, Iran.

## Patient’s History:

The patient was a 62-year-old man presenting to the emergency department 6 hours after the onset of dizziness, nausea and vomiting. The patient complained of numbness of the right side of her body and reported swallowing problems. The initial examination showed the patient was alert and stable. The left side of her face was sweating while the right side was completely dry. The neurological examination revealed the patient was alert, and the right pupil was about 2 mm smaller than the left eye pupil, and both pupils responded to light. A paresis was observed in the right side of the face, tongue and uvula. Uvula was slightly deviated to the right. Other signs included hoarseness and swallowing impairment. The muscle strength of all four limbs was 5/5. Babinski reflex was downward on both sides. The patient could not sit by herself, and leaned to the right.

**Figure 1:**
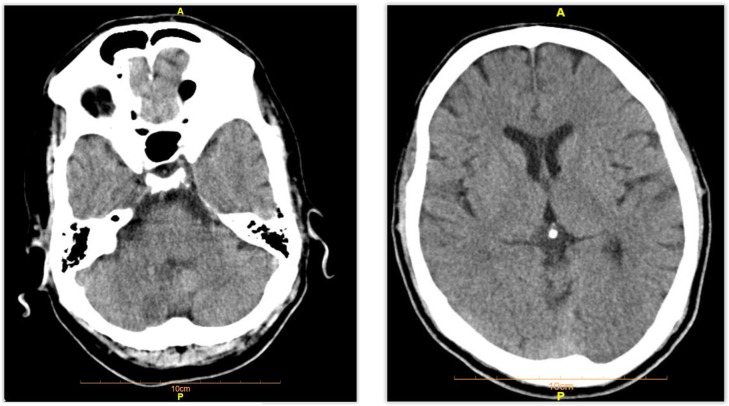
Axial Brain CT scan of the patient.

The patient had a history of primary coronary intervention (PCI) and stent placement four years ago. She had smoked a pack of cigarettes for 40 years. She used nitrocontin, pearl, lisinopril, carvedilol and furosemide. Laboratory tests were normal. The first CT scan in the emergency department was normal. As a brain stem infarction was suspected, MRI was performed and revealed an infarct (Figure 1). The patient received neurology consultation and was discharged with stable vital signs and the daily order of aspirin and atorvastatin after five days. The patient was asked to have weekly follow-up visits.

## Key questions:

- What are the radiologic findings in Figure 1?
- What is the importance of these findings?
- What other diagnostic modalities can be used for further assessment of this complication?

## Learning points:

- CT Scan Report:
  Both cerebral hemispheres appear normal with a suspicious hypoattenuation area in posterior inferior aspect of right cerebellar hemisphere.
- MRI Report:
  There are hyperintense lesions on T2WI/FLAIR sequences and mildly hypointense T1WI in right posterolateral medulla oblongata as well as the posterior inferior aspect of the right cerebellum in posterior inferior cerebellar artery (PICA) territory. These areas show restricted diffusion (high DWI and low ADC signal). These findings are in favor of acute infarction on the right posterolateral medulla oblongata as well as the posterior inferior aspect of the right cerebellum (Lateral medullary syndrome or Wallenberg syndrome).
  Wallenberg’s syndrome is defined as the posterior-inferior cerebellar artery infarction (PICA) or posterior-lateral medullary infarction (1). As a rare cerebrovascular disease, this syndrome accounts for 2.5% of ischemic attacks. The posterior-lateral region of medulla in the brain stem is affected (2, 3). Its most important risk factor is hypertension following smoking and diabetes (4). This syndrome is commonly prevalent in men in their sixty’s (5). This syndrome is often caused by thrombosis or embolism of vertebral artery. Other causes of the syndrome include bleeding, dissection of vertebral artery, cavernous angiomas, and tumors (6). The onset of this syndrome is abrupt. A precise neurological examination is a key to its diagnosis (2). Clinical signs and symptoms include dysphagia (due to the involvement of ambiguous nucleus), abnormal speech, ataxia, pain and numbness of the face (due to damage to CN VII and its nucleus) (7–9), vertigo and nystagmus (with nausea, vomiting and occasionally hiccups) secondary to infarction of vestibular nucleus or posterior-inferior cerebellum, diplopia and probably myoclonus of the upper jaw. Due to damage to the trigeminal nucleus, the patient experiences pain and absence of pupil reflex on the same side of the ischemia (7–9). Sensory defects of temperature and pain are seen in the trunk and the limbs in the opposite side of ischemia (3). The definitive or probable diagnosis is mainly obtained by physical examination and medical history. MRI is the best diagnostic test for confirmation of infarction in the inferior or lateral medullary regions of the brain. To find out the location of vascular occlusion and ruling out less frequent causes such as vertebral artery dissection, CT angiogram or MR angiogram are useful (10).
  Wallenberg syndrome is a type of ischemic stroke often caused by thrombosis or embolism in the heart or large arteries (11). The severity of clinical signs and emergency interventions and monitoring depend on the size and extent of the ischemic region (12). Previous studies have reported different types of visual impairments in these patients, such as Horner’s syndrome, diplopia, saccade, and nystagmus (13). Our case had a right eye ptosis, absence of sweating of the right side of the face, a smaller right eye pupil (Horner’s syndrome on the right side). Different degrees of dysphagia may be seen in 51% to 94% of the patients (12). Our case had slight hoarseness, paresis of upper jaw, right uvula and dysphasia. The patient complained about numbness and weakness of the right side of the face. The facial numbness is probably due to the involvement of the facial nerve fibers (15).

**Figure 2:**
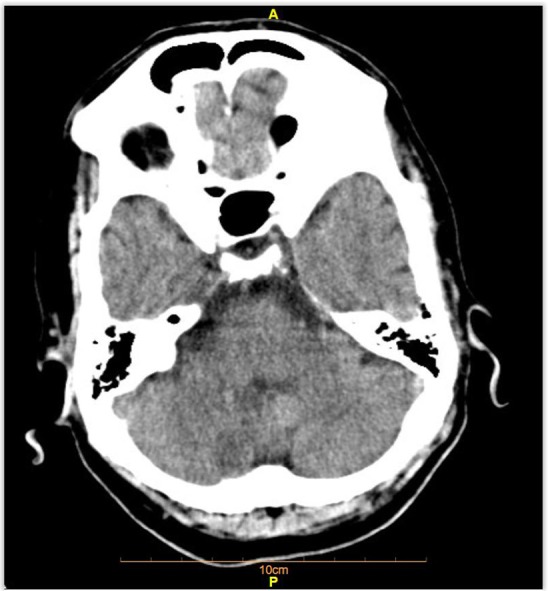
Axial Brain CT scan of the patient Suspicious hypo density focus in posterior inferior aspect of right cerebellar hemisphere

**Figure 2:**
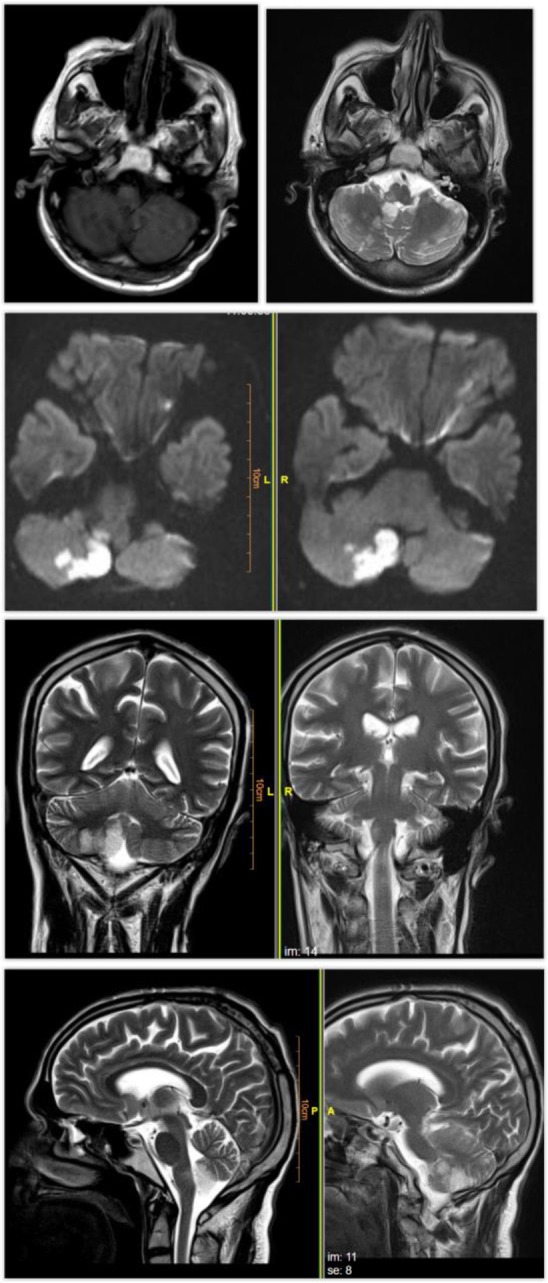
Brain MRI of the patient.
